# What Is the Cause of Appendicitis and Is the Disease on the Increase?

**Published:** 1912-02

**Authors:** Henry K. Leake

**Affiliations:** Dallas, Texas


### TEXAS MEDICAL JOURNAL.

Established July, 1885

F. E. DANIEL, M. D., - - - - Editor, Publisher and Proprietor

Published Monthly.—Subscription, $1.00 a Year.

VOL. XXVII. AUSTIN, FEBRUARY, 1912. No. 8.

The publisher is not responsible for the views of the contributors.

### Original Articles.

For Texas Medical Journal.

### What Is the Cause of Appendicitis and Is the Disease On the Increase?

BY HENRY K. LEAKE, A. M., M. D., DALLAS, TEXAS.

Following a delayed request of the chairman that I would read a paper in this section, it occurred to me that I might venture, in some measure at least, to answer a question so often asked of the physician and surgeon: What is the cause of appendicitis, and is the disease on the increase?

Concerning the latter part of the question, I assume that after the errors of diagnosis are considered, the consensus of belief is that the real disease is growing in frequency. In the light of our recent views on the pathology of appendicitis the first part of the question should take a different form: What are the causes of appendicitis? The answer to this, making an explanation to the laity if not to the professional man, is a very difficult one.

In my opinion, the chief cause of appendicitis is catarrh of the large bowel and that, owing to our present mode of life, this catarrh is on the increase. Affecting the mucous coat of the bowel, the disease need not be acute, although, as hinted by Moynihan, children, even below one year of age, frequently have appendicitis arising during the course of an entero-colitis; and that this appen-

*Read before the North Texas District Medical Society, December 14, 1911.

dicitis often escapes the diagnosis of the physician, who, impressed by the teaching of the text-books, is misled seriously and will give purgatives for abdominal pain and thus transform what might be a disease of mild character into one of a fatal tendency. Hence Moynihan's dictum: "Never give purgatives in a case of abdominal pain," and probably he intended to add, especially in the case of children; for at this time appendicitis may be the most important pathological factor in many of these little patients having gastric irritation and mucous discharges from the bowels, the catarrh of the bowel having passed rapidly within the lumen of the appendix in which condition laxatives or purgatives are highly dangerous. On the other hand, the disease oftener is in the chronic form; it may be latent, existing without the knowledge of the individual; a number of persons are the subjects of this latent form of bowel catarrh that finally extending into the appendix may exist here latent and thus prove the forerunner of an acute attack of appendicitis. Examples of this truth have been emphasized in my practice, but I refer not to them in detail. Also subacute cases of intestinal catarrh may have this ulterior termination. These observations are in line with those of Samuel Wilks of London, who many years ago insisted that appendicitis, after all, was but an original coecitis, and in his "Reminiscences," published recently, reiterates his former opinion, holding that the disease has been misnamed by the profession; an assertion that may be valuable in early directing the attention of physicians to the intestinal conditions that I have mentioned and probably by some means forestalling their end-results in appendicitis.

For both anatomical and physiological reasons, the disease in the bowel is found more intense in that portion from which the appendix has its origin, but it may begin and continue solely at this point. The inflammation spreading along the mucous surface may enter the estuary of the appendix and pass within to effect the lining of its narrow canal. There is a heaped-up fold of mucous membrane—the so-called Gerlach's valve—at the mouth of the appendix that may ineffectually guard the entrance; later the swelling of this, together with that of the inner wall of the appendix, blocks the way, thus preventing necessary drainage of the organ when an explosion of acute appendicitis may ensue, followed by the well known evil consequences. Particularly does this occur if there be kinking or angularity of the organ, or an embedding of this deep within the walls of the coecum. It should be realized that the acute attack is the end-result, not the beginning of the case of appendicitis; the catarrh is the antecedent, and in many cases remains after the appendix has been removed; although it can not be denied that this removal subsequently will effect beneficially the progress of the intestinal catarrh and render its treatment easier. These remarks might refer particularly to a case of chronic disease of an appendix having its origin abnormally distant from the ileo-coecal valve and lying, in coma vigil, behind the coecum in the subcoecal fossa, where the diseased organ may exist in this state for months or years, non-suppurating and giving little, if any, evidence of its presence until aroused into the acute stage of a lethal appendicitis, which even then in some patients is difficult of positive diagnosis that not the signs of Head, Morriss, Rovsing and Bastedo may determine.

Some time ago at a seaside resort in England and also at Harrowgate, this matter was investigated with the startling discovery that a large number of the community had this catarrh of the large bowel in a latent form, and many had appendicitis. The conclusion was reached that here cause and effect were well demonstrated. Bacterial decomposition of the food products is very liable in the large intestine, as has been pointed out by Mechnikoff, Harvey, Cushing, and other observers, and especially active in the coecum. This bacterial activity has been shown by Cushing to be increased by the use of purgatives, hence the deduction drawn from these researches by Moynihan in his denunciation of these agents when appendicitis is present. But inertia of the coecum, leading to stagnation of its contents and putrefaction, may initiate catarrh of the organ that precedes the inflammation of the appendix. That this coecal immotility is a prevalent condition among the masses at the present day will hardly be questioned, and this may be a principal factor in the numerous cases of constipation that are seen by the physician. The decomposition of food-residue in the coecum and other portions of the large bowel is favored and rendered more hurtful to the economy by the atmospheric changes occurring during the spring and autumn months. These changes are liable to throw out of balance the complementary relation between the action of the skin and that of the intestines, thereby forcing stress upon the latter, deranging secretion and digestion and causing bacterial virulence with the result—catarrh of the mucous surface that may superinduce a fatal degree of appendicitis.

So difficult is it to treat successfully these catarrhs of the large intestine and chronic constipation that this portion of the intestinal canal is placed under the ban, and by Lane and Mummery of England is eliminated by surgical operation either by extirpation or by joining the ilium to the large intestine low down in its continuity—a radical piece of surgery that must gain popularity slowly, if at all. Like the appendix, the large intestine is held to be not absolutely essential to the welfare of the economy and may become a standing menace to health and safety.

Appendicitis may be traced to a variety of causes. For example: In England it was alleged that eating foodstuffs out of tins, chiefly of American manufacture, was the cause of appendicitis. Innumerable and infinitesimal particles of the metal of the container on microscopical examinations were evident in the food mass. That being swallowed, these particles were carried within the appendix, producing minute erosions that offered a place of least resistance for the attack of intestinal bacilli, the result being a more or less destruction of the tissues of the appendicular wall. This plausible view made slight impression upon the profession. However, the old opinion that the presence of foreign bodies within the appendix is the sole cause of appendicitis has been abandoned, but the list of such cases is growing and will have greater prominence in the future.

It is stated that in Algiers, where very little meat is eaten, comparatively, appendicitis is rare, leading to the suggestion that appendicitis has close relation to the inordinate consumption of meat, which is the custom in other countries, such as Germany, England, France, and America. If there be any connection between the two it is not invariable, but circumstantial, although there is no doubt that meat eating in the countries mentioned is excessive, and, therefore, unnecessary for the wants of the system, but it is my observation that negroes that are large meat eaters are almost free from appendicitis; however, they enjoy robust digestion.

Confirming Chittenden, it is claimed by distinguished experimenters that 100 grammes of proteid should be consumed by the average person every twenty-four hours; of late it has been shown that the very best of health can be maintained on less than 75 grammes. Largely, meat is composed of the proteid element, that most important in the formation and repair of our bodies, but which, if taken in excess, or is of improper character, decomposes into poisonous by-products that are absorbed as toxins into the circulation and at the same time cause an intestinal catarrh that, coinciding with this absorption, may eventuate in appendicitis. More than this, the normal secretions of the intestines are altered, quantities of mucus are poured out into the lumen of the bowel inviting and causing growth and multiplication of bacteria that further add their poisonous excreta to the food mass that forms a culture medium for their development and attack upon the diseased inner coat of the bowel, the appendix participating as a climax of chemical and bacteriological pathology. Respecting this condition it should be remembered that atony of the coecum may be the primary and principal factor entailing constipation, and, notwithstanding the diatribe recently launched by Dr. Edwin Walker of Evansville, Indiana, against the common habit of using laxatives and purgatives, the practice will continue because it finds its demands in an almost universal aberrant physiology of the large intestine with deferred, it may be, but widespread pathology in the arteries and other organs of the system. That through ignorance a rational and salutary measure is abused is no reason why it should not be employed in suitable circumstances, and these circumstances are well known to be suitable in a large percentage of the community. According to Klemm, in a number of cases considered to be chronic appendicitis, appendectomy does not remove the difficulty; the disease in these instances consists of "atony of the coecum resulting in coprostasis and colonic catarrh. Coecal atony is the consequence of inflammatory changes in the coecal serosa which occur in attacks and are caused by retardation of the discharge of the secretion from the appendix into the coecum." I suggest that the sequence of events is just the reverse, that the inflammatory changes in the coecum are primary beginning in the mucosa and preceding and causing a retardation of the secretions of the appendix, which is a secondary factor, being in its turn the primary cause of an appendicitis that follows; but the removal of the appendix not always cures the inflammatory changes which linger in the walls of the coecum.

Donald Hood advanced the probability that appendicitis is caused by a specific germ as yet unknown; that this germ by evolution has become very virulent and is transferred by some means from one individual to another. Therefore, appendicitis is a contagious disease, or perhaps infectious through some germ carrier, and in this way its increase is accounted for. But Mantle explains that owing to our mode of life inducing intestinal catarrh the normal bacillus of the intestine develops an unnatural virulence so that contagion, strictly as such, need not be evoked to clear up the mystery of the acknowledged increase of the disease; nevertheless, he admits that infection is possible, as may be conjectured from the fact that several members of the same family, or persons living in close relation, may have appendicitis of the different types of severity.

About five years ago, Payne and Poynter of London, after the most painstaking researches, claimed that they discovered the germ that caused rheumatism, a certain kind of diplococcus, which they had found in the fluid of the joints of persons afflicted with this malady. This diplococcus being injected into rabbits almost invariably caused the heart, joint, pleural and pericardial changes known to exist in the human subject when so called rheumatism was present. Although this claim was accepted by some authorities, in my judgment, it gained not the attention it deserved. Further exhaustive researches by these gentlemen have confirmed their earlier statements and besides have shown that appendicitis by the introduction of this germ into the system is produced in addition to the other changes ordinarily seen in rheumatism. Consequently, without denying that there exist other causes for appendicitis, they aver that the rheumatic diplococcus may cause appendicitis through the blood stream, and so is the result of an infection. But Adrian, in 1901 had demonstrated that appendicitis could be effected easily by other germs as well, for example: the staphylococcus, streptococcus, pneumococcus, and bacillus coli that had been supplied to the blood by the intravenous method. From this it would seem that undoubtedly there is a causal relation between appendicitis and rheumatism, but this had been asserted long ago by Beverly Robinson and by Edward Janeway, who in their clinical experience believed that they had aborted appendicitis in its earliest stages by anti-rheumatic remedies—an appendicitis that had its origin in the peritoneal envelope of the organ.

In the *New York Medical Record,* December 9, 1911, Beverly Robinson writes: "In appendicitis the frequent underlying cause is rheumatism and the evidence pathologically, from even the late remarkable experimental proof of it by Poynton and Paine, is the accompanying local peritonitis which is often present.

"The localized peritoneal inflammation not infrequently starts, as I believe, the inflammation in the appendix and not the appendix the inflammation in the peritoneum.

"In either case, get rid, if possible, of the primary cause by diet, habits, regime, and the use of salicin or salicylate of soda, and the number of cases of appendicitis will greatly diminish.

"Institute rational, effective treatment at the beginning of an attack and fewer operations will be required.

"As to fulminating cases, operate immediately, or, preferably, as I believe, wait for one of two things: (1) Perforation; (2) local abscess. In either of these circumstances operation is surely indicated.

"It must be remembered, however, that acute general peritonitis does not invariably mean perforation, and as this is true, when no perforation exists, even immediate operation does not increase the chances of the patient's recovery as compared with those from wise medical treatment.

"Further, there are many cases of appendicitis confounded with, or preceded and caused by, colitis. If we get rid of the colitis the appendicitis, when it exists, will disappear also."

Appendicitis may co-exist with inflammatory or other disease of the right uterine adnexa; the diseased organs may be separate or adherent to each other. If separate, the association may be a mere coincidence, and if adherent, the organs originally were in contact or in proximity, the inflammation either peri-ovarian or peri-appendicular, spreading from one organ to the other. A simultaneous occurrence, therefore, of the diseases may be referred each to its own specific cause, or to a common cause operating through the blood current as suggested by the researches of Adrian that made probable a general systemic infection that might localize itself in this region of the body. To explain the facts it is not necessary to invoke the presence of infection by way of the vessels in the so-called Clado's ligament, which, in my view, has doubtful anatomy. It is noteworthy that appendicitis may co-exist with disease of the left uterine adnexa, but in what percentage of cases has not been ascertained. A deposit of tubercle, syphilis or cancer may involve the appendix in a more or less degree of inflammation, but this is of secondary importance, which is merged with that of these diseases that are arranged under their own specific classification and will not be discussed here, except to be reminded that the knowledge of these infections strongly corroborates the statements of Adrian heretofore mentioned.

Paradoxically speaking, it seems that one of the inevitable causes of appendicitis is the unnecessary operations performed based upon an erroneous diagnosis. Contrary to the statement of some surgeons, the diagnosis of appendicitis always is not easy and sometimes impossible; the disease may exist without any symptoms whatever. Moreover, doctors are human only; they have their ambitions and obsessions like other folk, and often rush an operation without prudent and conscientious discrimination. Differentiation is hurried and superficial. It is much as when several men are seen running down the street someone cries, "Stop thief!" everybody turns out in pursuit only to apprehend and pommel someone who had not committed the offense; the real culprit had been passed unawares. Such operations swell the statistical list, which to that degree is misleading, but the diagnosis in Missouri is not more astute than elsewhere.

The following conclusions seem warranted:

1. The causes of appendicitis are various, both in nature and virulence, and this may account for the different forms of the disease.

2. Catarrh of the intestine, either acute, subacute or latent, is the chief cause of appendicitis.

3. The latent form of this catarrh may not be disclosed by looseness of the bowels, but usually is associated with, if not caused by an alternate constipation or obstipation that is the initial factor in the train of events terminating in appendicitis; and that this fact misguides the physician that examines not the discharges from the rectum in patients with obscure symptoms.

4. Especially children with abdominal pain, vomiting and mucous stools, frequently are subjects of appendicitis; this should be excluded before the routine dose of calomel or castor oil be administered, such medication being unwise and often disastrous.

5. The first symptom of appendicitis is pain in the epigastrium and around the umbilicus, where it may remain for several hours previously to its final location in the right iliac region; hence purgatives should be withheld until the diagnosis of appendicitis is negatived or established, and in the latter case not to be employed before the performance of an operation. A simple enema to empty the rectum, if indicated, is sufficient and not hurtful.

6. The public should be warned against the evil effects of rapid, irregular, excessive eating and improper food, at the present so universal—the banquet table has precipitated attacks of acute appendicitis. Oral sepsis also, as shown by Hunter, may be the starting point of putrefaction down the intestinal tract, with far-reaching bad effects, both upon the mucosa and distant organs that may prove fatal to the individual, as witness the death of the editor of the *London Lancet* from this cause several years ago.

7. Probably rheumatism never expends itself upon the appendix only, but in diagnosis the favorite sites of this poison should be examined critically that this disease may be differentiated and thereby an operation perhaps avoided by remedies that are known to antagonize the rheumatic diplococcus circulating in the blood and tissues of the patient.

8. Pneumonia, particularly its basal form, is seen not infrequently to be associated with appendicitis and may progress insidiously; it is good practice in all cases of appendicitis to examine the lungs before submitting the patient to the depressing ordeal of an abdominal operation.

9. It is rational during the performance of an operation to observe closely the condition of the coecal walls as regards color, thickening and distension, for these may point to an existing catarrh that afterwards may persist. Furthermore, this has reference to prognosis that may connote dress, a proper food regimen, rest, and suitable laxatives or bowel irrigation for the complete relief of the patient.

10. During operation for the removal of the appendix in women, the pelvic organs should be examined always; the best incision for the purpose being that introduced by Battle of St. Thomas' Hospital, London; it yields a ready access, firm cicatrix, and, if desired, convenient drainage. Should extensive disease in the left half of the pelvis be discovered, an additional incision may be required. Moreover, through the Battle incision examination of the organs in the upper abdomen can be made.

11. Although Mellier in 1826, and Addison in 1836, wrote remarkably descriptive papers on inflammation of the appendix, American surgical genius has developed the diagnosis of and operation for appendicitis. I predict that the prevention of the disease and possibly its cure awaits the genius of American physicians, some of whom are devoting an earnest and intelligent thought to the solution of this problem.

